# Impact of Integrated Exercise Interventions on Muscle Mass and Functional Outcomes in Patients With Decompensated Liver Cirrhosis and Sarcopenia: A Systematic Review

**DOI:** 10.7759/cureus.111449

**Published:** 2026-06-24

**Authors:** Rushikesh S Patil, Poovishnu T Devi, Janhavee S Brahmadande, Kiran S Dhaygude

**Affiliations:** 1 Department of Cardiopulmonary Sciences, Krishna College of Physiotherapy, Krishna Vishwa Vidyapeeth (Deemed to be University), Karad, IND

**Keywords:** decompensated liver cirrhosis, functional capacity, health-related quality of life, muscle mass, sarcopenia

## Abstract

Sarcopenia is a common and serious complication in patients with decompensated liver cirrhosis and is linked with reduced muscle mass, impaired functional capacity, poor physical performance, and decreased quality of life. Exercise-based rehabilitation and nutritional interventions have emerged as important non-pharmacological approaches for improving clinical and functional outcomes in this population. This comprehensive analysis sought to appraise the outcome of integrated exercise training programs on muscle mass, functional capacity, and quality of life in sarcopenic individuals with decompensated liver cirrhosis. There was an extensive search of the literature using PubMed, Google Scholar, Scopus, ScienceDirect, and Web of Science, published between 2012 and 2026. Randomized controlled trials (RCTs), clinical trials, observational studies, cross-sectional studies, and meta-analyses focusing on exercise, rehabilitation, and nutritional interventions were included. Risk of bias was checked using the Risk of Bias 2 (RoB 2) tool and the Appraisal Tool for Cross-Sectional Studies (AXIS) according to the study design. A total of 10 studies were found. Findings demonstrated that integrated exercise interventions, in addition to aerobic workouts, resistance training, balance training, and home-based rehabilitation programs, significantly enhance muscle strength, functional performance, aerobic capacity, body composition, and health-related quality of life. Nutritional interventions, such as branched-chain amino acid supplementation, also showed positive effects on muscle mass and maintenance of liver function. Most of the studies demonstrated moderate to high caliber with minimal potential for bias. The review concludes that integrated exercise training programs are safe and effective for increasing muscle mass, functional capacity, and quality of life for sarcopenic individuals with decompensated liver cirrhosis. Combined rehabilitation and nutritional strategies may play a vital part in the multidisciplinary management of cirrhosis-associated sarcopenia.

## Introduction and background

Liver cirrhosis is a progressive and irreversible condition characterized by diffuse hepatic fibrosis and nodular regeneration, ultimately leading to impaired liver function and multiple systemic complications. Among individuals with decompensated liver cirrhosis, common complications include ascites, hepatic encephalopathy, variceal bleeding, and severe metabolic disturbances [1]. In recent years, increasing attention has been directed toward sarcopenia, a condition involving progressive loss of skeletal muscle mass, strength, and physical performance, which is highly prevalent in patients with advanced liver disease [2]. Sarcopenia in cirrhotic individuals results from a combination of factors such as chronic inflammation, malnutrition, hypermetabolism, hormonal imbalance, reduced physical activity, and impaired protein synthesis [3]. The presence of sarcopenia in decompensated liver cirrhosis is associated with poor prognosis, increased risk of hospitalization, reduced exercise tolerance, impaired functional independence, lower survival rates, and diminished health-related quality of life [4]. Functional limitations caused by muscle weakness and fatigue significantly affect the ability of patients to perform activities of daily living, thereby increasing physical dependency and psychological distress [5].

Conventional medical management primarily focuses on controlling hepatic complications; however, non-pharmacological strategies, such as exercise rehabilitation, are gaining importance in improving overall patient outcomes. Exercise interventions, including resistance training, aerobic conditioning, balance exercises, and functional training, have demonstrated beneficial effects on muscle strength, physical performance, and metabolic health in chronic disease populations. An integrated exercise training program combining these components may provide a comprehensive rehabilitative approach for sarcopenic individuals with decompensated liver cirrhosis by targeting muscle wasting, reduced endurance, and impaired functional capacity simultaneously [6].

Despite growing evidence supporting exercise-based rehabilitation in chronic liver disease, limited studies have specifically evaluated the effectiveness of integrated exercise programs in sarcopenic individuals with decompensated liver cirrhosis [7]. Furthermore, the impact of such interventions on muscle mass, functional capacity, and health-related quality of life remains inadequately explored in this vulnerable population [6,7].

Therefore, the present study aims to qualitatively synthesize and critically evaluate the existing evidence regarding the effects of integrated exercise training programs on muscle mass, functional capacity, and health-related quality of life in sarcopenic individuals with decompensated liver cirrhosis. As a qualitative synthesis rather than a quantitative systematic review or meta-analysis, this article focuses on identifying, comparing, and interpreting findings across the available literature. The findings may contribute to the development of evidence-based rehabilitation strategies aimed at improving physical function, independence, and overall quality of life in this patient population.

## Review

Study design

A systematic review was conducted according to evidence-based review methodology using randomized controlled trials, clinical trials, cross-sectional studies, meta-analyses, and observational studies. Risk of bias was assessed using the revised Cochrane Risk of Bias 2 (RoB 2) tool for randomized controlled trials [8] and the Appraisal Tool for Cross-Sectional Studies (AXIS) for cross-sectional studies [9]. The systematic review was registered with the International Prospective Register of Systematic Reviews (PROSPERO; Registration number: CRD420261424455; registered on 15 June 2026).

Search strategies

To find studies assessing the qualitative synthesis, impact of exercise training, dietary treatments, and rehabilitation programs on muscle mass, functional ability, and health-related quality of life in people with liver cirrhosis and sarcopenia, a thorough literature search was carried out. Articles published between 2012 and 2026 were found by searching electronic databases such as PubMed, Google Scholar, Scopus, ScienceDirect, and Web of Science. Medical Subject Headings (MeSH) terms and keywords like sarcopenia, liver cirrhosis, exercise training, resistance training, aerobic exercise, functional capacity, muscle mass, quality of life, six-minute walk test (6MWT), branched-chain amino acids, rehabilitation, and nutrition intervention were used in the search strategy. To properly combine search phrases, the Boolean operators "AND" and "OR" were employed. Additional pertinent publications were found by manually screening the reference lists of the chosen research. The review includes cross-sectional research, meta-analyses, clinical trials, randomized controlled trials, and observational studies that were published in English. For randomized controlled trials, the RoB 2 tool [8] was used to assess risk of bias; for cross-sectional research, the AXIS tool was used [9].​​​​​​

Inclusion criteria

Male and female volunteers between the ages of 30 and 50 years who had been diagnosed with liver cirrhosis and classified as Child-Pugh class B and C with scores between 7 and 15 were included in the study. The causes of liver cirrhosis included non-alcoholic steatohepatitis (NASH), chronic hepatitis B patients undergoing antiviral therapy with virological suppression, alcohol-related liver disease with at least a year of abstinence, and chronic hepatitis C patients with sustained virological response. The search was restricted to research published up until April 2026, and only articles found on digital platforms like Google Scholar, ResearchGate, PEDro, MEDLINE, and PubMed were taken into consideration.

Exclusion criteria

Exclusion criteria included individuals with liver cirrhosis linked to large esophageal or gastric varices, active cancer, such as hepatocellular carcinoma, cardiac conditions, such as coronary artery disease and cardiac arrhythmias, chronic renal failure necessitating dialysis, hematological disorders, such as hemoglobin levels below 10 g/dL or platelet counts below 50,000, acute or chronic infections, and chronic lung diseases, such as severe asthma or chronic obstructive pulmonary disease (COPD). Furthermore, the review did not include any publications that did not match the predetermined inclusion criteria for a thorough assessment of therapeutic interventions and diagnostic systems.

Quality assessment

Standardized risk of bias assessment techniques were used to evaluate the methodological quality of the included studies in accordance with the study design. The RoB 2 tool was used to analyze clinical trials and randomized controlled trials [8]. It evaluated bias resulting from the randomization procedure, deviations from intended interventions, missing outcome data, outcome assessment, and selection of reported outcomes [8]. The AXIS was used to evaluate cross-sectional and observational research. It analyzed study objectives, sample selection, measurement validity, statistical analysis, reporting quality, ethical considerations, and risk of non-response bias [9]. Although there were some issues with participant and assessor blinding, the majority of randomized controlled trials showed minimal risk in the randomization procedure and result reporting. Although there were some issues with representativeness and non-response bias, cross-sectional studies usually demonstrated a minimal risk of bias. In general, the included papers showed moderate to good methodological quality, making them appropriate for the systematic review.

Results

A total of 10 articles met the predefined eligibility criteria for this review, providing a comprehensive overview of current diagnostic frameworks and therapeutic interventions for patients with liver cirrhosis.

**Figure 1:**
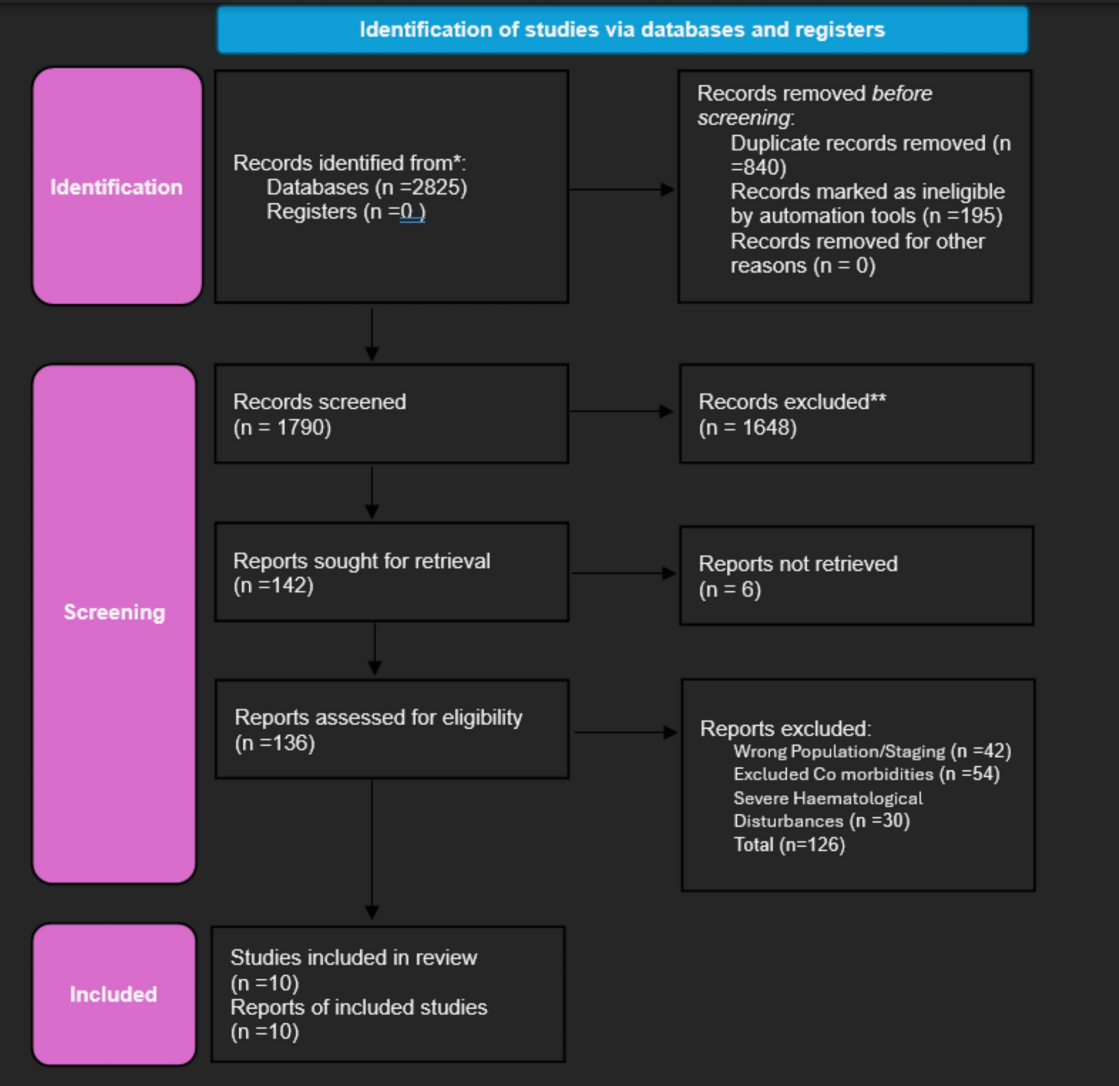
PRISMA flow diagram of study selection process for the systematic review.

The PRISMA flow diagram illustrating the study selection process for the systematic review is presented in Figure 1. A total of 2,825 records were identified through database searching, with no additional records obtained from registers. Before screening, 840 duplicate records and 195 records marked as ineligible by automation tools were removed, leaving 1,790 records for title and abstract screening. Out of these, 1,648 records were excluded. Full-text retrieval was attempted for 142 reports, of which six could not be retrieved. Subsequently, 136 reports were assessed for eligibility. Among these, 126 reports were excluded due to reasons including wrong population or staging (n = 42), exclusion of comorbidities (n = 54), and severe hematological disturbances (n = 30). Finally, 10 studies were included in the systematic review, as shown in Figure 1.

The included studies comprised randomized controlled trials, randomized clinical trials, cross-sectional studies, and a meta-analysis, with sample sizes ranging from 25 to 220 participants. The studies primarily evaluated the effects of exercise training, nutritional supplementation, resistance training, and combined multidisciplinary interventions on sarcopenia in individuals with liver cirrhosis and older adults [10]. Common outcome measures included muscle mass, muscle strength, physical performance, exercise capacity, quality of life, liver function, and functional capacity. Most interventional studies demonstrated significant improvements in muscle strength, aerobic capacity, balance, functional performance, and quality of life following exercise and nutritional interventions. Branched-chain amino acid supplementation was also found to improve muscle mass and liver function. Observational studies highlighted the association between sarcopenia and poorer physical function, reduced quality of life, and increased cardiovascular risk. Overall, the findings suggest that integrated exercise and nutritional interventions are safe and effective strategies for improving physical, functional, and clinical outcomes in sarcopenic individuals with liver cirrhosis, as shown in Table 1.

**Table 1:** Characteristics and summary of findings of included studies. 6MWT: six-minute walk test; BCAA: branched-chain amino acid; RCT: randomized controlled trial.

Author(s)	Year	Study design	Sample Size	Outcome measures	Interventions	Results	Conclusion
Zhang et al. [11]	2026	Randomized controlled trial	100	Nutritional parameters, balance ability	Comprehensive care with nutrition and exercise intervention	Combined intervention significantly improved nutrition status and balance ability.	Multidisciplinary management is effective in sarcopenic older adults
Brown et al. [12]	2025	Randomized clinical trial	38	Muscle strength, physical performance, exercise tolerance	Resistance training program	Resistance training significantly improved strength and physical performance.	Structured resistance training is safe and effective for cirrhotic patients
Hsieh et al. [13]	2024	Meta-analysis of randomized controlled trials	220	Body composition, exercise capacity, fatigue, quality of life	Analysis of exercise interventions	Exercise interventions improved exercise capacity, reduced fatigue, and enhanced quality of life.	Exercise therapy is beneficial for physical and functional recovery in liver cirrhosis.
Sirisunhirun et al. [14]	2022	Randomized controlled trial	40	Aerobic capacity, muscle mass, liver stiffness, spleen stiffness, and quality of life	12-week home-based exercise training program	Exercise improved aerobic capacity, muscle mass, and quality of life with a reduction in liver and spleen stiffness.	Home-based exercise is safe and effective in improving functional and clinical outcomes in cirrhosis patients.
Singh Tejavath et al. [15]	2021	Randomized clinical trial	106	Muscle mass, muscle strength, physical performance, survival, liver function	Branched-chain amino acid supplementation	BCAA supplementation improved muscle mass, strength, and maintenance of liver function.	BCAA therapy may help manage sarcopenia and improve prognosis in cirrhosis.
Román et al. [16]	2016	Randomized clinical trial	25	Functional capacity, body composition, fall risk	Structured exercise program	Significant improvement in functional capacity and body composition with reduced fall risk.	Exercise rehabilitation positively affects physical performance and safety in cirrhotic patients.
Beaudart et al. [17]	2015	Cross-sectional study	73	Quality of life, muscle strength, physical performance	Observational evaluation	Sarcopenia was linked with poorer quality of life and reduced physical function.	Sarcopenia negatively affects both physical and psychosocial health.
Vilaça et al. [18]	2013	Cross-sectional study	77	Body composition, muscle strength, 6-minute walk distance	Functional performance assessment	Better 6MWT performance was associated with greater muscle strength and healthier body composition	Functional exercise capacity reflects muscle health and physical fitness.
Tarantino et al. [19]	2012	Cross-sectional study	200	Carotid intima-media thickness, metabolic parameters	Clinical and metabolic assessment	Hepatic steatosis was associated with increased cardiovascular risk indicators.	Fatty liver disease may contribute to cardiovascular complications.

Assessment of Risk of Bias

Every selected study underwent a risk-of-bias assessment to guarantee the validity of the combined data. Using approved research methodologies, a thorough evaluation of the chosen literature was conducted to verify the reliability of the given results.

According to the RoB 2 tool's risk of bias assessment, the majority of the included randomized controlled trials had a low risk of bias due to the selection of reported findings, the randomization method, and missing outcome data. However, due to the challenge of blinding participants and assessors in exercise- and rehabilitation-based therapies, all studies raised some concerns about deviations from targeted interventions and outcome measures. In terms of overall risk of bias, all included randomized controlled trials were rated as having "some concerns," suggesting generally acceptable methodological quality with modest performance and detection bias problems. The cross-sectional studies that were considered had generally excellent methodological quality, according to the AXIS assessment [9]. Every study used proper study designs, appropriately specified the target population, applied appropriate sampling frames, employed validated outcome assessment instruments with appropriate statistical analyses, and explicitly expressed its goals and objectives. All research had sufficient descriptions of the procedures, findings, comments, ethical issues, and conflict-of-interest reporting. Nonetheless, certain issues with the selection process's representativeness and possible non-response bias were noted. Furthermore, none of the studies offered comprehensive information about non-respondents or a clear description of the steps taken to address them. Overall, there was little danger of bias in the included observational studies, with the main issues being selection and non-response bias, as shown in Tables 2-4.

**Table 2:** Risk of bias assessment of included studies using the RoB 2 tool. RoB 2: Risk of Bias 2.

Study	Bias arising from the randomization process	Bias due to deviations from intended interventions	Bias due to missing outcome data	Bias in the measurement of the outcome	Bias in the selection of the reported result	Overall risk of bias
Zhang et al. (2026) [11]	Low risk	Some concerns	Low risk	Some concerns	Low risk	Some concerns
Brown et al. (2025) [12]	Low risk	Some concerns	Low risk	Some concerns	Low risk	Some concerns
Sirisunhirun et al. (2022) [14]	Low risk	Some concerns	Low risk	Some concerns	Low risk	Some concerns
Singh Tejavath et al. (2021) [15]	Low risk	Some concerns	Low risk	Some concerns	Low risk	Some concerns
Román et al. (2016) [16]	Low risk	Some concerns	Low risk	Some concerns	Low risk	Some concerns

**Table 3:** Quality assessment of included cross-sectional studies using the AXIS tool. AXIS: Appraisal Tool for Cross-Sectional Studies.

AXIS appraisal question	Beaudart et al. (2015) [17]	Vilaça et al. (2013) [18]	Tarantino et al. (2012) [19]
Were the aims/objectives of the study clear?	Yes	Yes	Yes
Was the study design appropriate for the aims?	Yes	Yes	Yes
Was the sample size justified?	Yes	Some concerns	Yes
Was the target population clearly defined?	Yes	Yes	Yes
Was the sampling frame appropriate?	Yes	Yes	Yes
Was the selection process representative?	Some concerns	Some concerns	Some concerns
Were measures taken to address non-responders?	No	No	No
Were the outcome variables measured appropriately?	Yes	Yes	Yes
Were the tools piloted/published previously?	Yes	Yes	Yes
Was statistical significance clearly determined?	Yes	Yes	Yes
Were the methods described in enough detail?	Yes	Yes	Yes
Were the basic data adequately described?	Yes	Yes	Yes
Is there concern regarding non-response bias?	Some concerns	Some concerns	Some concerns
Was the info about non-respondents described?	No	No	No
Were the results internally consistent?	Yes	Yes	Yes
Were all method-defined results presented?	Yes	Yes	Yes
Were discussions and conclusions justified?	Yes	Yes	Yes
Were the study limitations discussed?	Yes	Yes	Yes
Were funding/conflicts of interest reported?	Yes	Yes	Yes
Was ethical approval/informed consent attained?	Yes	Yes	Yes

**Table 4:** Risk of bias assessment of the included meta-analysis using the ROBIS tool. ROBIS: Risk of Bias in Systematic Reviews.

Study	Domain 1: Study eligibility criteria	Domain 2: Identification and selection of studies	Domain 3: Data collection and study appraisal	Domain 4: Synthesis and findings	Phase 3: Overall risk of bias
Hsieh et al. (2024) [13]	Low risk	Low risk	Low risk	Low risk	Low risk

Discussion

The present systematic review evaluated the effects of integrated exercise training programs on muscle mass, functional capacity, and health-related quality of life in sarcopenic individuals with decompensated liver cirrhosis. The findings of this review demonstrate that structured exercise interventions, particularly when combined with nutritional support, produce significant improvements in muscle strength, aerobic capacity, physical performance, and overall quality of life in this patient population [11,20]. These findings support the growing evidence that rehabilitation-based strategies are both safe and beneficial in the management of cirrhosis-associated sarcopenia [21].

Sarcopenia is increasingly recognized as a major complication of decompensated liver cirrhosis due to its strong association with reduced survival, increased hospitalization, impaired mobility, and poor clinical outcomes [20,21]. The pathophysiology of sarcopenia in cirrhosis is multifactorial and includes chronic inflammation, hyperammonemia, hormonal imbalance, malnutrition, and physical inactivity [22,23]. As a result, interventions targeting both nutritional deficiencies and physical deconditioning are essential for improving patient outcomes. The present review found that integrated exercise programs incorporating resistance training, aerobic exercises, balance training, and functional rehabilitation effectively addressed these impairments [17-19].

The randomized controlled trials included in this review consistently demonstrated positive effects of exercise interventions on muscle mass and functional performance. Brown et al. (2025) reported significant improvements in muscular strength, exercise tolerance, and physical performance following a structured resistance training program in patients with liver cirrhosis [12]. Similarly, Román et al. (2016) observed improvements in body composition, functional capacity, and reduced risk of falls after participation in a supervised exercise program [16]. These findings suggest that resistance and functional training stimulate skeletal muscle adaptation and improve neuromuscular efficiency, thereby reducing physical frailty in cirrhotic individuals [24].

The findings of Sirisunhirun et al. (2022) further highlighted the effectiveness of a 12-week home-based exercise program, which improved aerobic capacity, muscle mass, and quality of life while also reducing liver and spleen stiffness. These results indicate that even home-based rehabilitation protocols may be feasible and effective in improving both physical and clinical outcomes in patients with cirrhosis [14]. Home-based interventions may also improve adherence and accessibility, particularly in individuals with limited mobility or frequent hospital visits [22]. Nutritional support also played an important role in improving sarcopenic outcomes. Singh Tejavath et al. (2021) demonstrated that branched-chain amino acid supplementation significantly improved muscle mass, muscle strength, and maintenance of liver function. Nutritional therapy combined with exercise may therefore produce synergistic effects by enhancing protein synthesis and reducing muscle catabolism [15,23,25]. Similarly, Zhang et al. (2026) found that comprehensive care involving exercise and nutritional intervention improved balance ability and nutritional parameters in sarcopenic individuals, emphasizing the importance of multidisciplinary management approaches [11].

The clinical utility of tracking these improvements is supported by Vilaça et al., who demonstrated that superior 6MWT performance directly correlates with greater muscle strength and healthier body composition. In sarcopenic cirrhotic patients where muscle wasting and fatigue severely limit mobility, utilizing objective tools like the 6MWT provides vital data on physical functioning and the overall success of exercise rehabilitation [18].

Furthermore, the systemic and multi-organ impact of advanced liver disease is highlighted by Tarantino et al., who linked hepatic steatosis with poor metabolic parameters and elevated cardiovascular risks. Because liver conditions drive widespread metabolic and circulatory changes alongside muscle catabolism, these findings emphasize the necessity of early detection and holistic, interdisciplinary care models that combine exercise rehabilitation with nutritional management [19].

The meta-analysis by Hsieh et al. (2024) provided strong evidence supporting exercise-based rehabilitation by demonstrating improvements in exercise capacity, fatigue, body composition, and health-related quality of life across multiple randomized controlled trials. Improved quality of life observed in several studies may be attributed to enhanced physical independence, reduced fatigue, greater social participation, and improved psychological well-being [13,20,26]. Beaudart et al. (2015) also reported that sarcopenia was strongly associated with poorer physical and psychosocial health, further supporting the need for rehabilitation-focused interventions [17].

Vilaça et al. (2013) found a strong correlation between body composition, muscle strength, and functional exercise ability in their cross-sectional investigation [18]. According to the study, those who performed higher on the 6MWT also had stronger muscles and a more balanced body. These results emphasize how crucial physical performance evaluation is as a measure of general muscle health and physical fitness [25]. Reduced exercise capacity is frequently linked to weariness, muscle atrophy, and diminished functional independence in people with long-term illnesses, including sarcopenia and liver cirrhosis [21]. As a result, evaluation instruments such as the 6MWT may offer useful data on physical functioning and the results of rehabilitation [25].

In the same direction, Tarantino et al. (2012) highlighted the link between elevated cardiovascular risk factors and hepatic steatosis. According to the scientists, poor metabolic parameters and increased carotid intima-media thickness were associated with fatty liver disease, suggesting a higher risk of cardiovascular problems [19]. These results imply that liver illness contributes to systemic metabolic and circulatory changes in addition to impairing hepatic function [22]. In order to lower long-term cardiovascular risks, the study emphasizes the significance of early detection and thorough therapy of metabolic abnormalities in people with liver disease. The idea that liver-related conditions are closely linked to decreased physical performance, changed body composition, and more systemic issues is supported by the collective findings of these investigations [23]. In order to improve overall patient outcomes, they further emphasize the necessity of interdisciplinary approaches that concentrate on exercise rehabilitation, nutritional management, and metabolic health [16,24].

Although the findings of this review are encouraging, several limitations should be considered. Considerable heterogeneity existed among the included studies with respect to exercise protocols, intervention duration, intensity, outcome measures, and patient characteristics [20,21]. Sample sizes in several trials were relatively small, limiting the generalizability of the findings. Additionally, blinding of participants and assessors was difficult in exercise-based interventions, resulting in some concerns regarding performance and detection bias [20]. Long-term follow-up data were also limited, making it difficult to determine the sustainability of the observed benefits [26].

Despite these limitations, the overall methodological quality of the included studies was moderate to good, and the majority demonstrated consistent positive outcomes associated with exercise rehabilitation [20,21]. The evidence suggests that integrated exercise training programs are safe, feasible, and effective for improving muscle mass, functional capacity, and health-related quality of life in sarcopenic individuals with decompensated liver cirrhosis [23,24,27].

Future research should focus on large-scale randomized controlled trials with standardized exercise protocols and long-term follow-up to establish optimal rehabilitation guidelines for this population [26]. Further studies exploring the combined effects of exercise, nutritional supplementation, and multidisciplinary rehabilitation approaches may provide additional insight into comprehensive management strategies for cirrhosis-associated sarcopenia [6,23,24].

## Conclusions

Integrated exercise training programs are effective in improving muscle mass, functional capacity, and health-related quality of life in sarcopenic individuals with decompensated liver cirrhosis. Exercise interventions such as resistance, aerobic, and functional training, especially when combined with nutritional support, help improve muscle strength, physical performance, and overall well-being. These findings support the inclusion of multidisciplinary rehabilitation programs in the management of sarcopenia associated with liver cirrhosis. This study emphasizes how combined exercise and nutritional rehabilitation can significantly improve the quality of life for those with decompensated liver cirrhosis. The incorporation of organized rehabilitation programs in normal clinical treatment is encouraged by the favorable results seen in all of the trials. Overall, the results provide patients with cirrhosis-associated sarcopenia hope for improved physical function, independence, and quality of life.
